# Bias Associated with Peripheral Non-Invasive Compared to Invasive Arterial Blood Pressure Monitoring in Healthy Anaesthetised and Standing Horses Using the Bionet BM7Vet

**DOI:** 10.3390/vetsci9020052

**Published:** 2022-01-28

**Authors:** Shaun Pratt, Tamsin S. Barnes, Nicholas Cowling, Karla de Klerk, Joanne Rainger, Albert Sole-Guitart, Solomon Woldeyohannes, Wendy Goodwin

**Affiliations:** 1 School of Veterinary Science, Gatton Campus, The University of Queensland, Gatton 4343, Australia; t.barnes@uq.edu.au (T.S.B.); n.cowling@uq.edu.au (N.C.); k.deklerk24@gmail.com (K.d.K.); j.rainger@uq.edu.au (J.R.); a.guitart@uq.edu.au (A.S.-G.); s.woldeyohannes@uq.edu.au (S.W.); w.goodwin@uq.edu.au (W.G.); 2 Queensland Alliance for Agriculture and Food Innovation, The University of Queensland, Gatton 4343, Australia

**Keywords:** ABP, NIBP, IBP, horse, anaesthesia, Bionet BM7Vet

## Abstract

To compare arterial blood pressure (ABP) measured invasively (IBP) to ABP measured non-invasively (NIBP) via oscillometry in healthy anaesthetised and standing horses using the Bionet BM7Vet. Fourteen horses were anaesthetised for elective procedures (anaesthetised group) and 10 horses were enrolled for standing blood pressure manipulation (standing group). In both groups, IBP and NIBP-corrected to heart level were measured every 3 min using the Bionet BM7Vet. The overall mean difference (bias), standard deviation and limits of agreement (LOA) were calculated for paired IBP and NIBP systolic (SAP), mean (MAP) and diastolic (DAP) blood pressure measurements. In anaesthetised horses, the NIBP cuff was placed at either the proximal tail base or the metacarpus. Invasive MAP was used to retrospectively characterise measurements into hypotensive (≤70 mm Hg), normotensive (71–110 mm Hg) or hypertensive (≥111 mm Hg) subgroups. In standing horses, the NIBP cuff was placed at the tail base only and invasive MAP was manipulated to achieve hypertension (≥126 mm Hg) and hypotension (≤90 mm Hg) using phenylephrine and acepromazine, respectively. When measuring NIBP at the tail in anaesthetised horses, the Bionet BM7Vet failed on 8/185 occasions and overestimated SAP, MAP and DAP during hypotension and normotension. The biases (lower, upper LOA) for MAP were −11.4 (−33.3, 10.5) and −6.0 (−25.8, 13.8) mm Hg, respectively. Hypertension could not be evaluated. When measuring NIBP at the metacarpus in anaesthetised horses, the Bionet BM7Vet failed on 24/65 occasions and underestimated SAP, MAP and DAP when all ABP subgroups were combined. The bias (lower, upper LOA) for pooled MAP was 3.6 (−44.3, 51.6) mm Hg. When measuring NIBP at the tail in standing horses, the Bionet BM7Vet failed on 64/268 occasions and underestimated SAP, MAP and DAP during hypotension, normotension and hypertension. The biases (lower, upper LOA) for MAP were 16.3 (−10.5, 43.1), 16.6 (−19.5, 52.7) and 30.0 (−8.1, 68.0) mm Hg, respectively. Monitoring NIBP on the Bionet BM7Vet in anaesthetised horses overestimated ABP at the tail and underestimated ABP at the metacarpus. The device inaccurately detected hypotension and should be used cautiously. In standing horses, the Bionet BM7Vet underestimated ABP at the tail, especially during pharmacologically induced hypertension.

## 1. Introduction

Arterial blood pressure (ABP) measurements provide important cardiovascular information and are especially useful in hemodynamically unstable and anaesthetised patients [1,2,3,4]. In horses, ABP measured invasively (IBP) is considered the gold standard ABP measurement technique. An arterial catheter is connected via non-distensible, heparinised tubing to a transducer placed at the phlebostatic axis. Pressure wave changes within the transducer transmit electrical signals for interpretation and display on the patient side monitor. Despite arterial catheters being relatively simple to place in anaesthetised horses in hospital, for the monitoring of standing sedated, critically ill and anaesthetised horses in the field, the risk for contamination, arteritis and equipment failure is high [5]. Consequently, devices measuring ABP non-invasively might be beneficial in horses.

Devices which measure ABP non-invasively (NIBP) are routinely used in human and small domestic animal patients, however, inaccuracy and poor reliability compared with IBP mean they remain underused in horses. Oscillometric NIBP devices utilise a peripherally placed arterial bladder cuff to record pressure wave oscillations induced by blood flow turbulence during vascular release. The monitor measures MAP at maximal oscillation amplitude, then uses proprietary algorithms to calculate SAP and DAP. Veterinary specific oscillometric devices have been studied in conscious and anaesthetised adult horses [6,7,8,9,10,11,12] and foals [13,14] across variably defined hypotensive and hypertensive ABP states. Estimates for accuracy and reliability vary considerably in these studies, with the underestimation and overestimation of ABP by the non-invasive method reported. Additionally, as mathematical algorithms used for oscillometric calculation are monitor specific, inter-monitor extrapolation is inappropriate.

The accuracy and precision of oscillometric NIBP monitoring using the Bionet BM7Vet has not been evaluated in horses. The purpose of this study was to compare ABP measured invasively and non-invasively in anaesthetised and standing horses to provide recommendations on the clinical use of the Bionet BM7Vet. It was hypothesised that the greatest variability in accuracy and precision would occur during hypotension and hypertension.

## 2. Materials and Methods

All procedures were performed with approval of The University of Queensland’s Production and Companion Animal Ethics Committee (SVS/449/17). The study enrolled two populations of horses; client-owned horses anaesthetised for elective surgery (observational study) and an experimental group of university-owned horses for standing blood pressure manipulation.

### 2.1. Anaesthetised Group

#### 2.1.1. Animals

Fourteen client-owned horses presenting for surgery at The University of Queensland’s Equine Specialist Hospital were enrolled into the observational study arm. Demographic data included 9 Thoroughbreds, 2 Quarter Horses, 1 Arabian, 1 Australian Stock Horse and 1 Irish Sport Horse (10 females, 4 males). Horses were aged (mean ± SD) 22 ± 20 months and weighed 370 ± 107 kg at the time of surgery. Procedures included arthroscopic surgery for osteochondritis dissecans (tarsus *n* = 6, stifle *n* = 3, fetlock *n* = 1), wound exploration (*n* = 1) and transphyseal bridging of the distal radius (*n* = 3). All horses were an American Society of Anaesthesiologists (ASA) classification I or II based on the results of a physical examination and pre-anaesthetic biochemical and haematological analysis. Animals were housed in individual stalls for varying durations dependant on their medical care requirements. Water was available ad libitum and grassy lucerne was provided morning and night.

#### 2.1.2. Anaesthesia

The skin overlying the left jugular vein was aseptically prepared and following the subcutaneous deposition of 20 mg lidocaine 2% (Lignocaine 20; Troy Laboratories, NSW, Australia), a 14 G, 3-inch catheter (BD Angiocath; Becton Dickinson Rowa Australia, QLD, Australia) was placed for the delivery of all intravenous (IV) agents. Anaesthetic technique was at the discretion of the primary anaesthetist. Thirteen horses were premedicated with medetomidine (Medetomidine; Troy Laboratories, NSW, Australia) 5 μg/kg IV, methadone (Methone; Ceva Animal Health, NSW, Australia) 0.1 mg/kg IV and acepromazine (Acemav-10; Mavlab, QLD, Australia) 0.02 mg/kg IV. General anaesthesia was induced 5 to 10 min later with ketamine (Ketamil; Troy Laboratories, NSW, Australia) 2.2 mg/kg IV and midazolam (Hypnovel; Roche Products, NSW, Australia) 0.06 mg/kg IV. One horse was premedicated with butorphanol (Butogesic; Troy Laboratories, NSW, Australia) 0.04 mg/kg IV, xylazine (Xylazil-100; Troy Laboratories, NSW, Australia) 0.5 mg/kg IV and medetomidine 1 μg/kg IV and was induced with propofol (Fresofol; Fresenius Kabi, NSW, Australia) 2 mg/kg IV.

Once recumbency was achieved and orotracheal intubation performed, all horses were hoisted onto a padded equine surgical table and positioned either in dorsal (*n* = 12) or lateral (*n* = 2) recumbency in the surgical suite. Partial IV anaesthesia was maintained with a medetomidine (2–3.5 μg/kg/hr IV) infusion (CRI) and either isoflurane (Isofol; Zoetis Australia, NSW, Australia) (*n* = 10) or sevoflurane (Sevorane; AbbVie, NSW, Australia) (*n* = 4) in oxygen. A large animal ventilator (Mallard; Mallard Medical/AB Medical Technologies, CA, United States of America) was used for controlled ventilation and lactate ringers’ solution (Compounds Sodium Lactate; Multipoint Technologies, VIC, Australia) was provided at approximately 5 mL/kg/hr IV throughout anaesthesia. A dobutamine (Dobutamine; Sandoz, NSW, Australia) infusion (0.25–1 μg/kg/min IV) was available to rectify trending- or to treat hypotension if required.

#### 2.1.3. Catheterisation and Invasive Blood Pressure Apparatus

Following the aseptic preparation of the skin, a 20 G, 1.4 inch catheter (Optiva; Smiths Medical International, Lanks, United Kingdom) was placed into either the facial artery (*n* = 13) or transverse facial artery (*n* = 1) and connected via a non-distensible tubing to a disposable transducer and a pressurised (300 mm Hg), heparinised 1 L saline bag (2 IU/mL heparin in 0.9% saline). The transducer (Disposable Blood Pressure Transducer; Unimed Medical Supplies, SZ, China) was placed at the level of the right atrium (sternal manubrium for lateral recumbency and humeral tuberosity for dorsal recumbency). The transducer was connected via a transducer cable to the Bionet BM7Vet and was calibrated (zeroed) to atmospheric pressure. The IBP system was assessed for accuracy by comparing the pressures with those concurrently measured using a manual mercury manometer pressure system (Mercurial Sphygmomanometer, A.C. Cossor & Son Surgical Ltd, London). Both systems were pressurized, and agreement between the transducer and the manometer was confirmed at pressures of 0, 50, 100, 150 and 200 mm Hg.

#### 2.1.4. Non-Invasive Blood Pressure Apparatus

The NIBP arterial cuff was placed at the most proximal section of the tail base with the bladder centred over the middle coccygeal artery during all periods of anaesthesia (*n* = 14). Following approximately 10 recordings and if it did not impose on the sterile field, the cuff was moved to the distal metacarpus with the bladder centred over the medial and lateral palmar metacarpal arteries (*n* = 9). If in lateral recumbency, the non-dependant limb was used. The cuff size was determined to be approximately 40% of the appendage circumference and was connected directly to the Bionet BM7Vet, set to large cuff size and to auto inflate to maximum pressure.

#### 2.1.5. Arterial Blood Pressure Assessment

Cardiorespiratory variables and clinical observations of anaesthetic depth were monitored per hospital protocol. The Bionet BM7Vet device was used to simultaneously record IBP and NIBP readings every 3 min. The IBP was noted at the completion of the NIBP cycle or when the NIBP device displayed an error message. The IBP trace was visually assessed every 3 min and described as good (prominent dicrotic notch with optimal dynamic pressure oscillations following fast flush) or poor (overdampened or underdampened). If poor, the invasive tubing was inspected for line abnormalities and the apparatus flushed.

Arterial blood pressure state classification in anaesthetised horses was extrapolated from previous studies [6,7,9,11,15,16] and can be found in Table 1.

### 2.2. Standing Group

#### 2.2.1. Animals

Ten mares (5 Standardbreds, 1 Stockhorse, 1 Warmblood, 1 Warmblood × Thoroughbred, 1 Stockhorse × Standardbred and 1 Spanish Horse × Thoroughbred) belonging to The University of Queensland’s Research Herd were enrolled into the experimental arm. These horses were aged 175 ± 40 months and weighed 505 ± 37 kg. Horses were yarded in small group paddocks the day prior to the study and monitored for 24 h following study completion. On the day of the study, all horses were classified as an ASA I based on the results of a physical examination. Ad libitum access to pasture and water was provided.

#### 2.2.2. Catheterisation and Invasive Blood Pressure Apparatus

Animals were restrained in stocks for data recording. The skin overlying the left jugular vein was catheterised as per the anaesthetised group. The skin overlying an easily palpable, peripheral artery of the head was aseptically prepared prior to the liberal application of topical anaesthetic cream (EMLA; AstraZeneca, NSW, Australia). Once 20 min had elapsed, a 20 G, 1.4-inch BD Angiocath was placed (transverse facial artery *n* = 6, facial artery *n* = 3) for IBP monitoring and the system was assembled as per the anaesthetised group. The transducer was placed at the level of the right atrium (humeral tuberosity) and linearity to the same mercury manometer pressure system was assessed as per the anaesthetised group.

#### 2.2.3. Non-Invasive Blood Pressure Apparatus

The NIBP device was assembled as per the anaesthetised group. The NIBP cuff was placed at the most proximal tail base with the bladder centred over the middle coccygeal artery for all recordings in all horses. To habituate horses to its pressurisation, the cuff was inflated through 5 cycles prior to data recording.

#### 2.2.4. Blood Pressure Manipulation

Arterial blood pressure state classification in standing horses was extrapolated from previous studies [10,17] and can be found in Table 1.

Following a minimum of 10 paired normotensive ABP measurements per horse, phenylephrine hydrochloride (Neosynephrine; CU Medical, CA, USA) 1–5 μg/kg/min IV was infused to induce hypertension for a maximum of 30 min. The infusion was immediately stopped if IBP SAP ≥ 175 mm Hg. Once MAP had returned to within 20 mm Hg of normotension, a bolus of acepromazine 0.05 mg/kg IV was administered. A second bolus was administered to a combined maximum dose of 0.1 mg/kg IV if hypotension did not occur within 20 min. If marked hypotension occurred (IBP MAP < 60 mm Hg), lactate ringers’ solution at 10 mL/kg IV was provided via a pressurised giving set and a dobutamine infusion was available.

Patients were monitored at 5 min intervals for pharmacological side-effects, including sinus bradycardia, increased perspiration, agitation, piloerection and second degree atrial-ventricular block during induced hypertension and sinus tachycardia, depression, weakness, and ataxia during induced hypotension.

### 2.3. Data Management and Statistical Analysis

To account for the effects of hydrostatic pressure on the position of the NIPB cuff, the difference (Δ) in vertical distance between the transducer and the NIBP cuff was calculated (in both anaesthetised and standing horses). A correction factor was applied to the NIBP data (0.078 mm Hg × Δ mm) [18] and only these values are here presented in the study. Data points were excluded from the analysis when the pulse rate measured using the NIBP method differed by ≥20% to the pulse rate on the IBP trace or heart rate on the ECG trace; whichever was found to be manually accurate.

Horse demographic data and SAP, MAP and DAP measurements made using each method (IBP and NIBP) were summarised using descriptive statistics. Non-normally distributed data was summarised as median (minimum–maximum) and normally distributed data as mean ± SD. Trajectory plots for paired SAP, MAP and DAP measurements were created for each horse and the data was checked for outliers.

Arterial blood pressure data comprised linked replicate measures, in that for each horse, the first replicate measure using the IBP method was recorded simultaneously with the first replicate measure using the NIBP method. Consequently, a proportion of variation between replicated measurements within horses was common to both methods. To account for repeated measurements and the linked nature of the data, methods for ABP assessment were compared using linear mixed models, with random effects fitted for method-by-horse interaction and horse-by-replicate interaction [19]. For each dataset, separate models were fitted for paired SAP, MAP and DAP values and Bland–Altman plots were used to visualise the results. Bias (IBP—NIBP), standard deviation (SD) and limits of agreement (LOA) were calculated from the model output. A positive bias denotes an underestimation of ABP by the NIBP method, whereas a negative bias denotes an overestimation of ABP by the NIBP method. LOA were derived from the 95% confidence intervals of repeat mean bias measurements on the same animal.

In reference to the only veterinary specific guidelines for IBP and NIBP evaluation [20] accuracy standards were defined as bias estimates < 10 mm Hg and precision standards as SD estimates < 15mm Hg for SAP, MAP and DAP.

Models were fitted using the MethComp package in R [21]. Stata/SE 15.1^®^ was used for all other analyses (Stata Statistical Software; Stata Corporation, College Station, TX, USA)

## 3. Results

**Figure 1:**
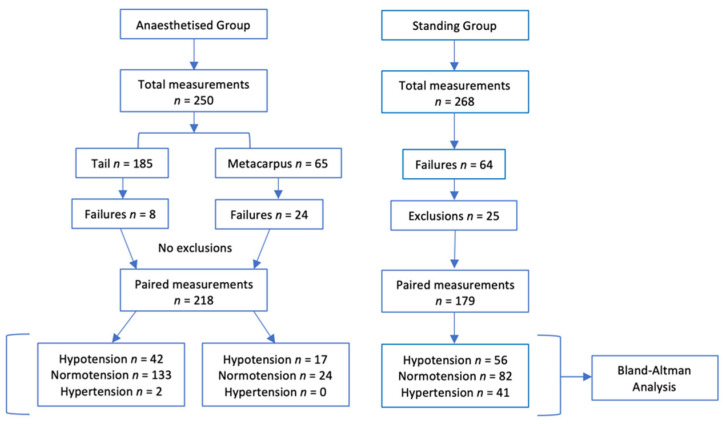
Flow diaphragm for comparing arterial blood pressure measured non-invasively to arterial blood pressure measured invasively using the Bionet BM7Vet in 14 healthy anaesthetised and 9 standing horses. Failures occurred when the Bionet BM7Vet failed to generate a non-invasive arterial blood pressure measurement by the completion of the inflation cycle. Paired measurements were excluded from the analysis when the pulse rate measured by the non-invasive method differed by ≥20% to the true pulse rate. Anaesthetised group: hypotension ≤ 70 mm Hg, normotension 71–110 mm Hg, hypertension ≥ 111 mm Hg. Standing group: hypotension ≤ 90 mm Hg, normotension 91–125 mm Hg, hypertension ≥ 126 mm Hg.

A flow diaphragm the results is presented in Figure 1.

### 3.1. Anaesthetised Group

Anaesthesia was maintained for a median duration of 66 (30–87) min without complication in all horses. Dobutamine was required in 9 horses for a median duration of 39 (6–54) min per horse. A total of 250 data points were collected during the observational period: including 185 from the tail and 65 from the metacarpus. The NIBP device failed 8/185 (4.3%) at the tail (*n* = 1 hypotension, *n* = 7 normotension) and 24/65 (36.9%) at the metacarpus (*n* = 9 hypotension, *n* = 15 normotension). The remaining 218 valid, paired ABP measurements were included in the statistical analysis. From the tail, this included 42 paired readings collected from 9 horses during hypotension (IBP MAP 64.5 (54–70) mm Hg) and 133 paired readings collected from 14 horses during normotension (IBP MAP 81 (71–110) mm Hg). From the metacarpus, this included 17 paired readings collected from 5 horses during hypotension (IBP MAP 68 (63–70) mm Hg) and 24 paired readings collected from 8 horses during normotension (IBP MAP 77.5 (71–92) mm Hg). Only 2 hypertensive measurements were collected from a single horse at the tail, meaning hypertension could not be analysed separately.

**Figure 2:**
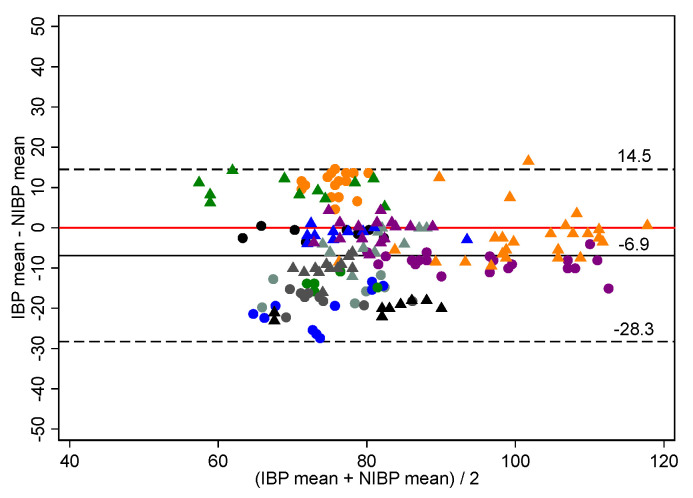
Bland–Altman plot of agreement for 177 paired mean arterial blood pressure measurements (mm Hg) made non-invasively compared to those made invasively using the Bionet BM7Vet in 14 healthy, anaesthetised horses. The non-invasive arterial blood pressure cuff was placed at the proximal tail base. Repeated observations from the same horse are plotted using the same colour-shape combination. The solid black line indicates mean bias, and the dashed lines indicate lower and upper limits of agreement. The solid red line represents zero bias. IBP invasive blood pressure, NIBP non-invasive blood pressure. Bias defines accuracy.

**Figure 3:**
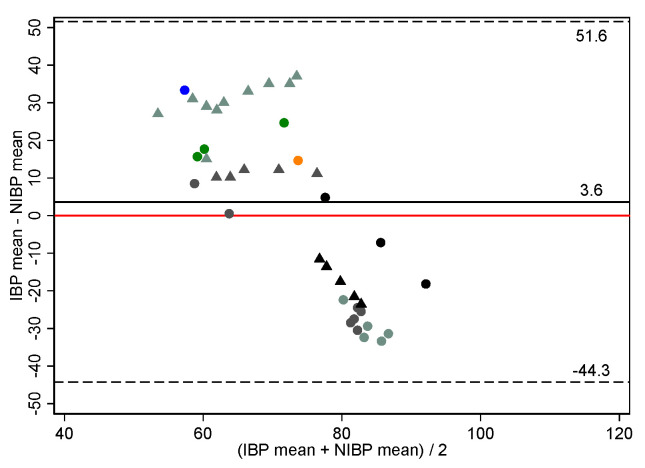
Bland–Altman plot of agreement for 41 paired mean arterial blood pressure measurements (mm Hg) made non-invasively compared to those made invasively using the Bionet BM7Vet in 9 healthy, anaesthetised horses. The non-invasive arterial blood pressure cuff was placed at the metacarpus. Repeated observations from the same horse are plotted using the same colour-shape combination. The solid black line indicates mean bias, and the dashed lines indicate lower and upper limits of agreement. The solid red line represents zero bias. IBP invasive blood pressure, NIBP non-invasive blood pressure. Bias defines accuracy.

Individual horse trajectory plots (Supplementary Figure S1) indicated trends of IBP-NIBP variation differed between NIBP cuff location. As a result, paired ABP measurements were separated by NIBP location (tail, metacarpus) for the Bland–Altman analysis presented in Table 2, Figure 2 and Figure 3 and Supplementary Figures S2 and S3. Due to the limited number of paired ABP measurements, metacarpal data remained pooled for the statistical analysis.

When measuring NIBP at the tail, mean biases for SAP, MAP and DAP were negative for all subsets: indicating NIBP measurements were generally overestimates of ABP. In comparison, when measuring NIBP at the metacarpus, mean biases for pooled SAP, MAP and DAP were positive: indicating NIBP measurements were generally underestimates of ABP.

### 3.2. Standing Group

A total of 268 data points were collected from 9 conscious horses. One horse was withdrawn from the study following two failed arterial catheterisation attempts. Hypertension and hypotension were achieved in all 9 horses. The NIBP device failed on 64/268 (23.9%) occasions. This included 5/62 (8.1%) failures during hypotension, 11/100 (11%) failures during normotension and 48/106 (45.3%) failures during hypertension. The ≥20% pulse rate-based exclusion criteria removed a further 25 of the remaining 204 data points (12.3%). This included 1/57 (1.8%) during hypotension, 7/89 (7.9%) during normotension and 17/58 (29.3%) during hypertension. The remaining 179 valid, paired ABP measurements were included in the statistical analysis. This included 56 paired readings collected during hypotension (IBP MAP 78 (59, 90) mm Hg), 82 paired readings collected during normotension (IBP MAP 110 (91, 125) mm Hg) and 41 paired readings collected during hypertension (IBP MAP 146 (126, 182) mm Hg). During the phenylephrine infusion, second degree atrial-ventricular block and piloerection were identified in 5 horses and all 9 horses experienced agitation (trunk swaying, weight shifting, vocalisation), increased perspiration and sinus bradycardia. All horses experienced sinus tachycardia during induced hypotension. Following collection of the final hypotensive measurement, one horse developed pronounced hypotension, weakness, and depression (acepromazine 0.1 mg/kg IV was administered in total). This horse required fluid support and additional close monitoring for a short period, however, made a full recovery.

**Figure 4:**
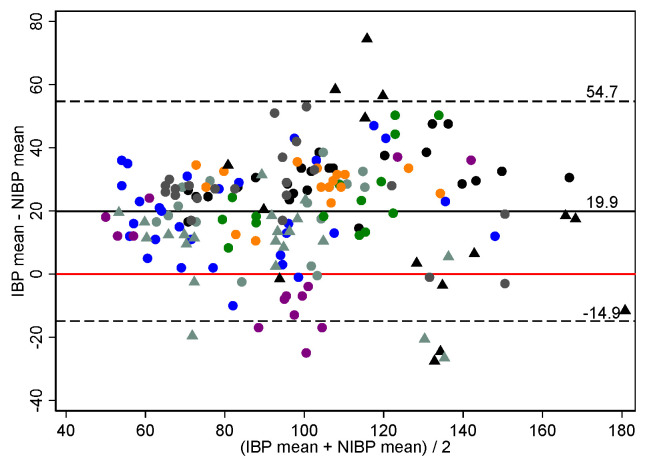
Bland–Altman plot of agreement for 179 paired mean arterial blood pressure measurements (mm Hg) made non-invasively compared to those made invasively using the Bionet BM7Vet in 9 healthy, standing horses. The non-invasive arterial blood pressure cuff was placed at the proximal tail base. Repeated observations from the same horse are plotted using the same colour-shape combination. The solid black line indicates mean bias, and the dashed lines indicate lower and upper limits of agreement. The solid red line represents zero bias. IBP invasive blood pressure, NIBP non-invasive blood pressure. Bias defines accuracy.

The Bland–Altman analysis for standing horses is presented in Table 3, Figure 4 and Supplementary Figure S4. When measuring NIBP at the tail, mean biases for SAP, MAP and DAP were positive for all subsets; indicating NIBP measurements were generally underestimates of ABP.

## 4. Discussion

This study compared ABP measured invasively to ABP measured non-invasively via oscillometry in separate populations of anaesthetised and standing horses using the same Bionet BM7Vet multi-parameter monitor. In anaesthetised horses, MAP and DAP measured non-invasively at the tail met accuracy (bias; MAP-6 mm Hg, DAP-4 mm Hg) and precision standards (SD; MAP 9.9 mm Hg, DAP 9.7 mm Hg) during normotension, however, failed to meet accuracy standards during hypotension (bias ≥ 10 mm Hg). Bias and SD estimates for SAP were inferior to MAP and DAP generally. Arterial blood pressure measured non-invasively at the metacarpus remained pooled for the statistical analysis due to a limited sample size. Pooled NIBP data measured at the metacarpus met accuracy standards (bias: SAP 9.0 mm Hg, MAP 3.6 mm Hg, DAP 0.8 mm Hg), yet, failed to meet precision standards (SD ≥ 15 mm Hg). In standing horses, NIBP measured at the tail failed to meet accuracy standards during hypotension, normotension or hypertension (bias ≥ 10 mm Hg) and only MAP and DAP met precision standards during hypotension (SD; MAP 13.4 mm Hg, DAP 14.3 mm Hg).

To provide recommendations on the clinical use of the Bionet BM7Vet, NIBP agreement to IBP during periods of hypotension and hypertension were of particular interest. The ideal monitor would generate NIBP measurements accurate and precise in their approximation of ABP. Arterial blood pressure measured non-invasively at the tail inaccurately detected hypotension in anaesthetised and standing horses, being an overestimation and underestimation of ABP, respectively. Hypertension could only be assessed in standing horses, where NIBP inaccurately and imprecisely underestimated ABP. These results support our hypothesis and align with the company’s product recommendations: “non-invasive blood pressure monitoring is not recommended for animals with hypotension, hypertension, arrhythmias or extremely high or low heart rate. The software algorithm cannot accurately compute NIBP or animals with these conditions” [22]. Hypotension remains a common morbidity in equine anaesthesia [4] and while hypertension is less common, equine metabolic syndromes, tourniquet use and chronic or severe pain (laminitis) are known to cause hypertension in anaesthetised and conscious horses [23,24]. Arterial blood pressure measured invasively, therefore, remains the standard for monitoring anaesthetised patients and tailoring therapeutics to cardiovascular-unstable patients. Clinical decision making based on NIBP is cautioned.

Mean arterial blood pressure is the average arterial pressure over the cardiac cycle and most reliably indicates central tissue perfusion pressure [17]. Clinicians use MAP to adjust interventional therapies and deliver anaesthetic agents appropriately, especially, where cardiac output and micro perfusion (lactate, base excess, oxygen extraction) monitoring is unavailable. Measuring NIBP at the tail in anaesthetised horses during normotension, was the only subset where MAP satisfied both accuracy (bias—6.0 mm Hg) and precision (SD 9.9 mm Hg) standards simultaneously. Estimates of bias and SD for MAP generally worsened during hypotension (anaesthetised and standing horses) and hypertension (standing horses). This trend of IBP-NIBP variability dependant on ABP state classification (hypotension, normotension or hypertension), is inconsistently reported within the literature. Certain manuscripts describe suboptimal NIBP agreement during hypotension [9,11], while others report good agreement in adults [6,12] and foals [13,14]. Until a monitor can reliably generate accurate NIBP estimates of ABP, the use of this monitoring modality will remain underused in horses.

The inferior precision estimates for NIBP measured at the metacarpus compared with the tail in anaesthetised horses, may reflect greater limb manipulation during surgery or poorer cuff contact. The proximal tail base has historically been used for NIBP studies in horses [6,7,8,9,10,11,12,13,14], and is preferable for its even contouring, limited compression during recumbency and limited manipulation during surgery. Despite returning accurate under-estimates of ABP, NIBP measured at the metacarpus was significantly imprecise (SD > 22 mm Hg). The effects of cuff location and patient recumbency on NIBP approximation of ABP has been investigated previously. Drynan, Schier and Raisis [6] found poorer agreement in dorsally recumbent horses generally yet were unable to isolate a cuff-recumbency effect. Hatz, Hartnack, Kummerle, Hassig and Bettschart-Wolfensberger [8] concluded that the accuracy of the Sentinel device was optimised with the cuff placed over the coccygeal artery (tail base) in lateral recumbency and over the palmar digital artery (metacarpus) or dorsal metatarsal artery (metatarsus) in dorsal recumbency. However, their methodology did not correct for hydrostatic pressure; being the force acting on a column of blood due to the weight of the blood above it [18]. Hydrostatic pressure is zero as blood drains into the right atrium, providing a reference point for correct placement of the arterial transducer (phlebostatic axis) and being the basis for NIBP data correction (0.078 mm Hg × Δ mm) [18]. Despite incorporating a hydrostatic correction factor in the current study, NIBP devices are often placed over peripheral arteries unaligned with the right atrium, complicating the translation of results into clinical settings. Regardless, measuring NIBP with the Bionet BM7Vet at the metacarpus in anaesthetised horses is cautioned.

Oscillometric devices may incorrectly identify pulse flow in certain situations, for example, when the rate of cuff deflation to patient heart rate is too great, during arrythmias or in situations of reduced peripheral blood flow [25,26]. Many of the standing horses developed sinus bradycardia and second-degree atrioventricular block; likely the result of phenylephrine induced hypertension. The alpha-1 adrenergic alterations in peripheral blood flow may explain the reduction in MAP accuracy (Δ 13.4 mm Hg) between normotension and hypertension. Non-invasive blood pressure failure rates for standing horses are not reported in the literature, however, patient compliance, agitation, movement, and muscle tone, likely contributed to the greater rate of measurement failures in standing (64/268, 23.9%) compared with anaesthetised (32/250, 12.8%) horses, especially during periods of induced hypertension (48/106, 45.3%). In comparison, acepromazine induced hypotension in standing horses, may have improved flow volume through the coccygeal and metacarpal arteries and, thus, reduced NIBP failures in this subgroup (5/62, 8.1%).

A medetomidine infusion was delivered to all anaesthetised horses to provide intra-operative analgesia and improve anaesthesia recovery quality. Its impact on the detection of peripheral pulse flow using the non-invasive method is unconfirmed [8] and is difficult to predict, given alpha-2 agents are biphasic in their haemodynamic effects. Previous reports of NIBP failure rates in anaesthetised horses vary between monitors [7,8] and, between pharmacologically altered ABP states (hypotension, normotension and hypertension) [11].

The 20% pulse rate-based exclusion criterion excluded 25/204 (12.3%) data points from standing horses, including a large proportion of hypertensive measurements 17/58 (29.3%). It is likely that these excluded measurements would show greater variability than those which met the inclusion criteria; meaning the estimates of precision (SD and LOA) derived from the Bland–Altman analysis are likely underestimates of their true value. It is also possible that these excluded measurements may have underestimated or overestimated ABP to a larger degree than those which met the inclusion criteria; meaning the bias estimates may also be underestimates of their true value. Unfortunately, there is a lack of standardised exclusion (or inclusion) criteria used in IBP-NIBP comparative studies [6,7,9], and this complicates how clinicians interpret these studies.

Using the Bionet BM7Vet, Jacobs-Fohrman et al. [27] compared ABP measured invasively to ABP measured non-invasively in anaesthetised bitches undergoing elective ovariohysterectomy. They found that NIBP underestimated SAP, MAP and DAP during hypotension, normotension and, with the greatest variability, hypertension. The guidelines for IBP-NIBP evaluation [20] were satisfied by SAP, MAP and DAP during hypotension and MAP and DAP during normotension. These results are similar to other studies using veterinary specific multi-parameter monitors in dogs [28,29], with estimates for bias and SD comparably more accurate and precise than those reported in horses.

This study has limitations involving sample size and limited replicate measurements. It is statistically preferrable to use fewer replicate measurements collected from a larger number of horses, than to use more replicate measurements collected from a small number of horses. Access to large numbers of horses is always a challenging factor in veterinary research, however, this is the first study to investigate ABP in anaesthetised and standing horses (pharmacologically manipulated) using the Bionet BM7Vet.

Vasopressor and ionotropic therapies are frequently administered within a multi-modal anaesthetic approach to standing and anaesthetised horses. Their summative effect on oscillometric peripheral blood flow detection is largely uncategorised. The co-administration of dobutamine in 9/14 horses (being at the discretion of the primary anaesthetist) better reflects clinical situations, however, does introduce variability. Finally, ABP state manipulation to hypotension and hypertension in standing horses was achieved pharmacologically. How the accuracy and precision of the Bionet BM7Vet may differ during haemodynamic crises is unknown.

## 5. Conclusions

In conclusion, monitoring ABP via non-invasive oscillometry on the Bionet BM7Vet in anaesthetised horses overestimated ABP at the tail and underestimated ABP at the metacarpus. The device inaccurately detected hypotension at the tail when evaluated against the accepted veterinary guidelines and, therefore, should be used cautiously. In standing horses, the Bionet BM7Vet underestimated ABP at the tail, especially during pharmacologically induced hypertension. High failure rates discourage monitoring NIBP at the metacarpus in anaesthetised horses and at the tail in conscious horses with the Bionet BM7Vet.

## Figures and Tables

**Table 1:** Arterial blood pressure state classification.

**Anaesthetised Group** Hypotension IBP MAP ≤ 70 mmHg Normotension IBP MAP (71–110) mm Hg Hypertension IBP MAP ≥ 111 mm Hg
**Standing Group** Hypotension * IBP MAP ≤ 90 mm Hg Normotension IBP MAP (91–125) mm Hg Hypertension ^#^ IBP MAP ≥ 126 mm Hg

IBP arterial blood pressure measured invasively, MAP mean arterial blood pressure. * Induced with acepromazine 0.05–0.1 mg/kg intravenous. ^#^ Induced with phenylephrine hydrochloride 1–5 mcg/kg/m intravenous constant rate infusion.

**Table 2:** Bland–Altmann analyses for 218 paired arterial blood pressure measurements made non-invasively compared to those made invasively using the Bionet BM7Vet in 14 healthy, anaesthetised horses.

		No. Paired Observations	No. Horses	Bias (mm Hg)	SD (mm Hg)	LOA-L (mm Hg)	LOA-U (mm Hg)
**Proximal Tail Base**							
Systolic	Pooled	177	14	−12.9	14.1	−41.0	15.2
	Hypotension	42	9	−15.4	13.9	−43.1	12.4
	Normotension	133	14	−12.8	14.4	−41.5	16.0
	Hypertension	2	1				
Mean	Pooled	177	14	−6.9	10.7	−28.3	14.5
	Hypotension	42	9	−11.4	11.0	−33.3	10.5
	Normotension	133	14	−6.0	9.9	−25.8	13.8
	Hypertension	2	1				
Diastolic	Pooled	177	14	−5.4	10.3	−25.9	15.2
	Hypotension	42	9	−10.2	10.4	−30.9	10.6
	Normotension	133	14	−4.0	9.7	−23.4	15.4
	Hypertension	2	1				
**Metacarpus**							
Systolic		41	9	9.0	22.1	−35.1	53.2
Mean		41	9	3.6	24.0	−44.3	51.6
Diastolic		41	9	0.8	26.0	−51.1	52.7

No. number, SD standard deviation, LOA limits of agreement, U upper and L lower, MAP mean arterial pressure. Bias defines accuracy and SD defines precision. The non-invasive arterial blood pressure cuff was placed either at the proximal tail base or the metacarpus. Combined recordings on the proximal tail base (pooled) were separated into hypotension (MAP ≤ 70 mm Hg), normotension (71 ≤ MAP ≤ 110 mm Hg) and hypertension (MAP ≥ 111 mmHg).

**Table 3:** Bland–Altmann analyses for 179 *paired* arterial blood pressure measurements made non-invasively compared to those made invasively using the Bionet BM7Vet in 9 healthy, standing horses.

Parameter	Tension State	No. Paired Observations	No. Horses	Bias (mm Hg)	SD (mm Hg)	LOA-L (mm Hg)	LOA-U (mm Hg)
Systolic	Pooled	179	9	15.7	23.0	−30.4	61.7
	Hypotension	56	8	10.7	17.9	−25.1	46.6
	Normotension	82	9	14.4	24.4	−34.5	63.3
	Hypertension	41	9	23.7	24.1	−24.5	71.8
Mean	Pooled	179	9	19.9	17.4	−14.9	54.7
	Hypotension	56	8	16.3	13.4	−10.5	43.1
	Normotension	82	9	16.6	18.0	−19.5	52.7
	Hypertension	41	9	30.0	19.0	−8.1	68.0
Diastolic	Pooled	179	9	18.4	18.2	−18.0	54.7
	Hypotension	56	8	17.3	14.3	−11.3	46.0
	Normotension	82	9	14.3	18.1	−21.8	50.4
	Hypertension	41	9	26.2	22.9	−19.6	71.9

No. number, SD standard deviation, LOA limits of agreement, U upper and L lower, MAP mean arterial pressure. Bias defines accuracy and SD defines precision. The non-invasive arterial blood pressure cuff was placed at the proximal tail base. Combined recordings (pooled) were separated into hypotension (MAP ≤ 90 mm Hg), normotension (91 ≤ MAP ≤ 125 mm Hg) and hypertension (MAP ≥ 126 mmHg). Hypotension was induced with acepromazine 0.05–0.1 mg/kg intravenous and hypertension was induced with phenylephrine hydrochloride 1–5 mcg/kg/m intravenous constant rate infusion.

## Data Availability

Datasets analysed in this study are available upon request from the corresponding author. The data is stored on UQRDM and mediated access is available upon request.

## Supplementary Materials

The following supporting information can be downloaded at: https://www.mdpi.com/article/10.3390/vetsci9020052/s1, Figure S1. Individual horse trajectory plots for 218 paired mean arterial blood pressure (MAP) measurements made non-invasively (NIBP) compared to those made invasively (IBP) using the Bionet BM7Vet in 14 healthy, anaesthetised horses. The non-invasive arterial cuff was placed either at the proximal tail base or the metacarpus. Figure S2. Bland–Altman plots of agreement for 177 paired (a) systolic and (b) diastolic arterial blood pressure measurements (mm Hg) made non-invasively compared to those made invasively using the Bionet BM7Vet in 14 healthy, anaesthetised horses. The non-invasive arterial cuff was placed at the proximal tail base. Repeated observations from the same horse are plotted using the same colour-shape combination. The solid black line indicates mean bias, and the dashed lines indicate lower and upper limits of agreement. The solid red line represents zero bias. Figure S3. Bland–Altman plots of agreement for 41 paired (a) systolic and (b) diastolic arterial blood pressure measurements (mm Hg) made non-invasively compared to those made invasively using the Bionet BM7Vet in 9 healthy, anaesthetised horses. The non-invasive arterial cuff was placed at the metacarpus. Repeated observations from the same horse are plotted using the same colour-shape combination. The solid black line indicates mean bias, and the dashed lines indicate lower and upper limits of agreement. The solid red line represents zero bias. Figure S4. Bland–Altman plots of agreement for 179 paired (a) systolic and (b) diastolic arterial blood pressure measurements (mm Hg) made non-invasively compared to those made invasively using the Bionet BM7Vet in 9 healthy, standing horses. The non-invasive arterial cuff was placed at the proximal tail base. Repeated observations from the same horse are plotted using the same colour-shape combination. The solid black line indicates mean bias, and the dashed lines indicate lower and upper limits of agreement. The solid red line represents zero bias.
