# Supplementary material for: Bias Associated with Peripheral Non-Invasive Compared to Invasive Arterial Blood Pressure Monitoring in Healthy Anaesthetised and Standing Horses Using the Bionet BM7Vet
Source: Vet Sci. 2022 Jan 28;9(2):52. doi: 10.3390/vetsci9020052 (PMC8878245; doi:10.3390/vetsci9020052)
Supplement: Supplementary file 1 [file vetsci-09-00052-s001.zip › vetsci-1534983-supplementary.pdf]

SUPPLEMENTARY DATA

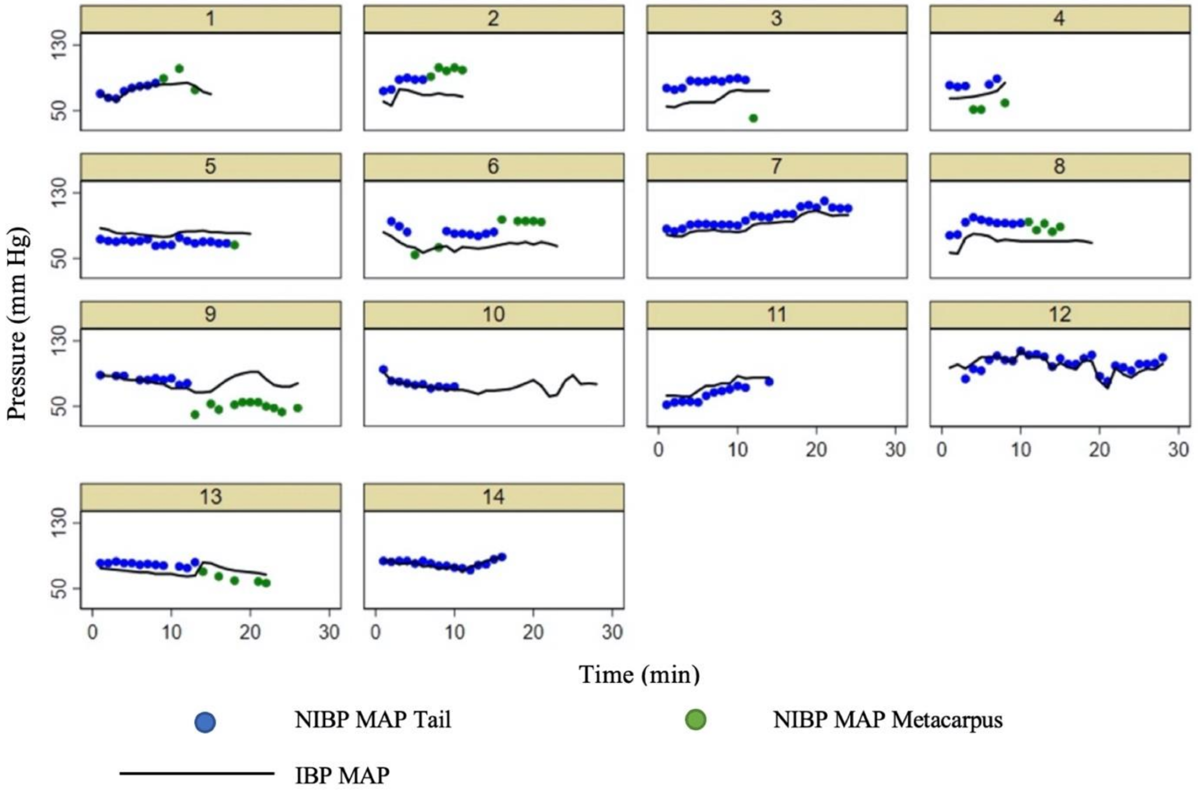

Figure S1: Individual horse trajectory plots for 218 paired mean arterial blood pressure (MAP) measurements made non-invasively (NIBP) compared to those made invasively (IBP) using the Bionet BM7Vet in 14 healthy, anaesthetised horses. The non-invasive arterial cuff was placed either at the proximal tail base or the metacarpus.

(a)

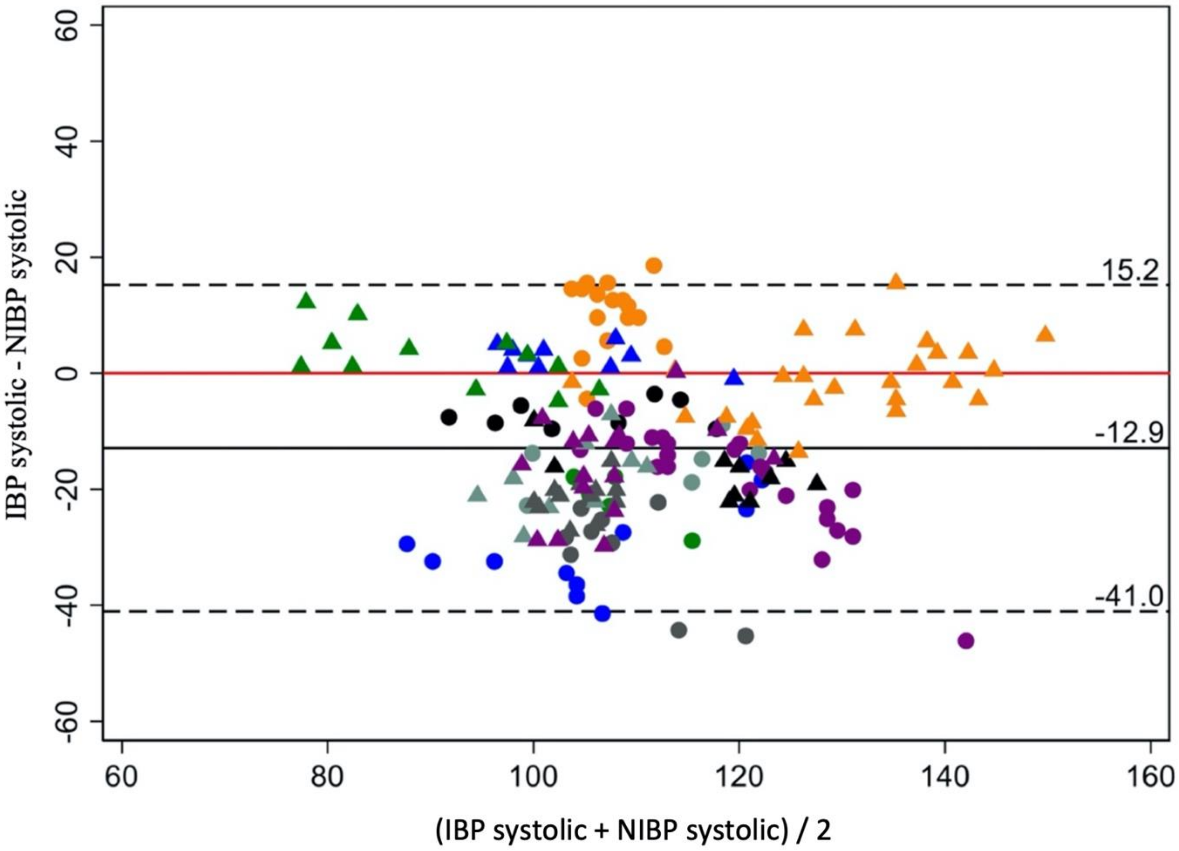

IBP invasive blood pressure, NIBP non-invasive blood pressure. Bias defines accuracy and SD defines precision.

(b)

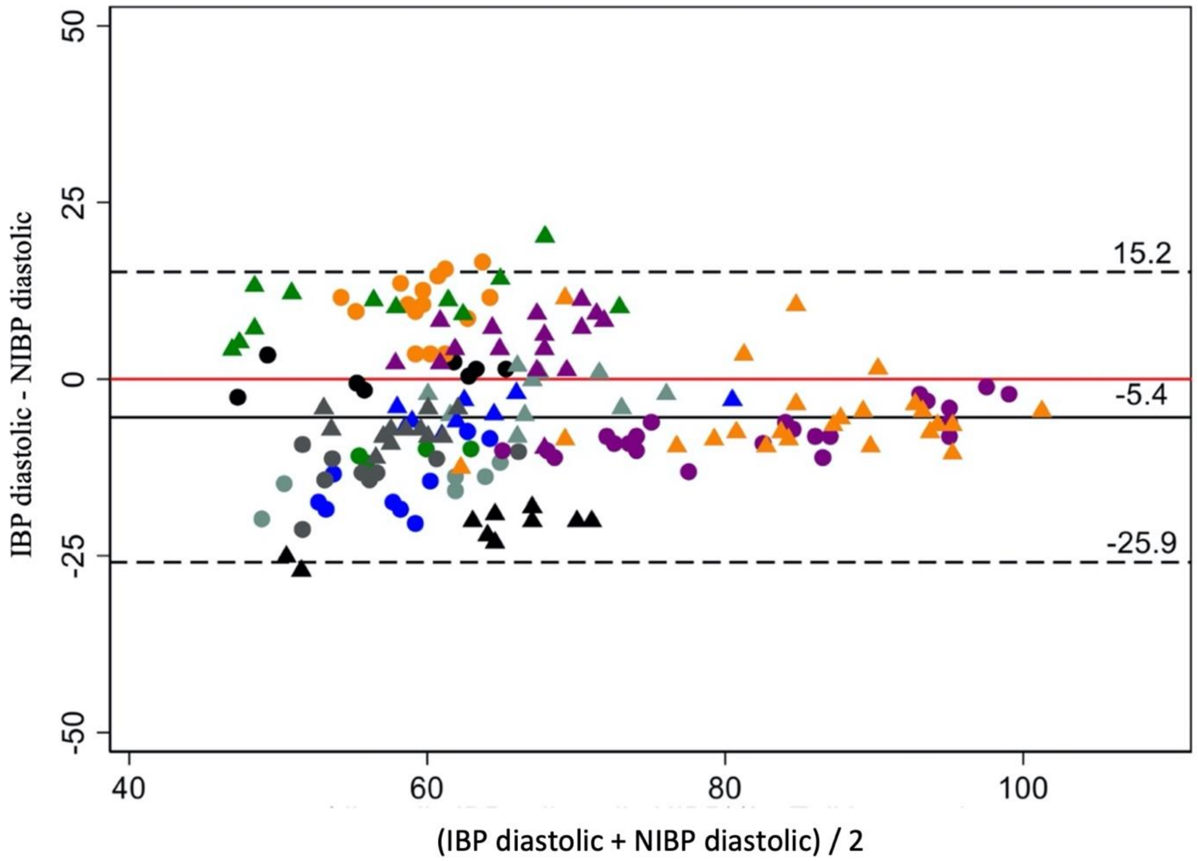

IBP invasive blood pressure, NIBP non-invasive blood pressure. Bias defines accuracy and SD defines precision.

Figure S2: Bland–Altman plots of agreement for 177 paired (a) systolic and (b) diastolic arterial blood pressure measurements (mm Hg) made non-invasive compared to those made invasively using the Bionet BM7Vet in 14 healthy, anaesthetised horses. The non-invasive arterial cuff was placed at the proximal tail base. Repeated observations from the same horse are plotted using the same colour-shape combination. The solid black line indicates mean bias, and the dashed lines indicate lower and upper limits of agreement. The solid red line represents zero bias.

(a)

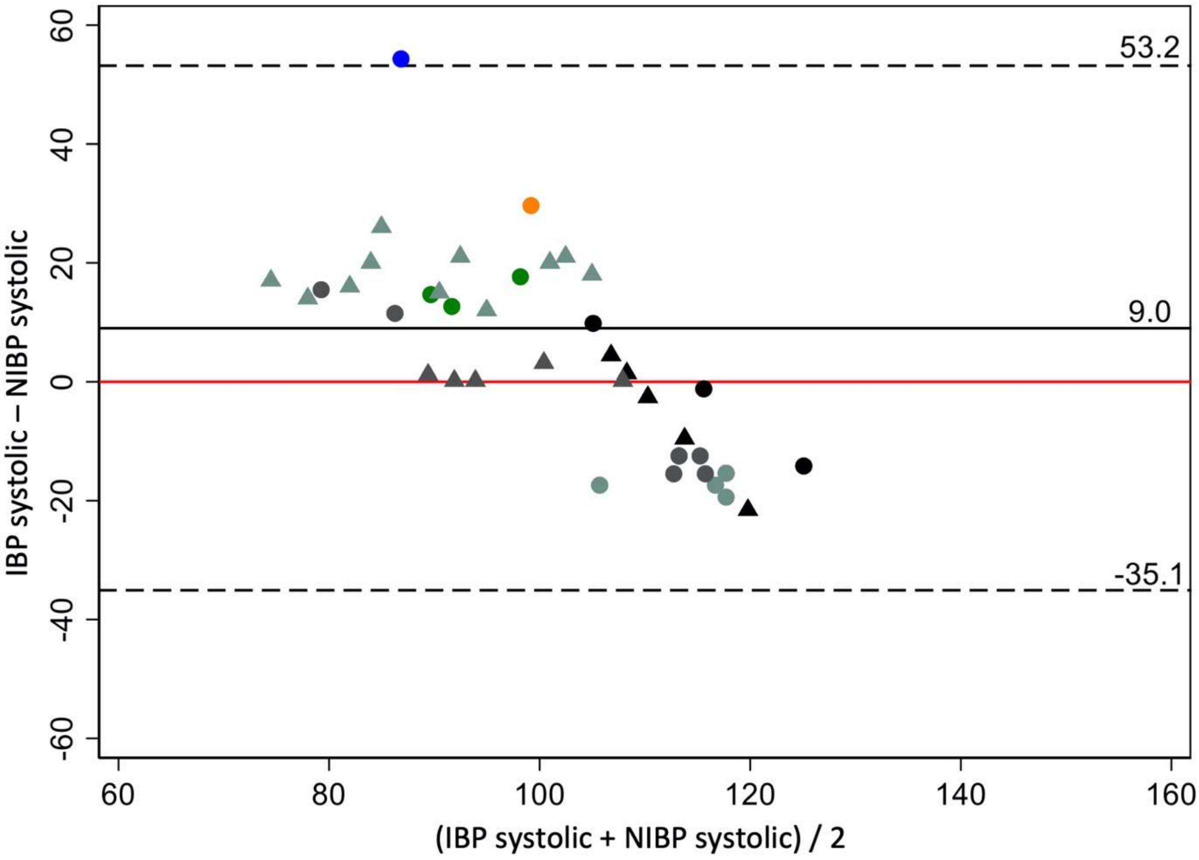

IBP invasive blood pressure, NIBP non-invasive blood pressure. *Bias* defines accuracy and *SD* defines precision.

(b)

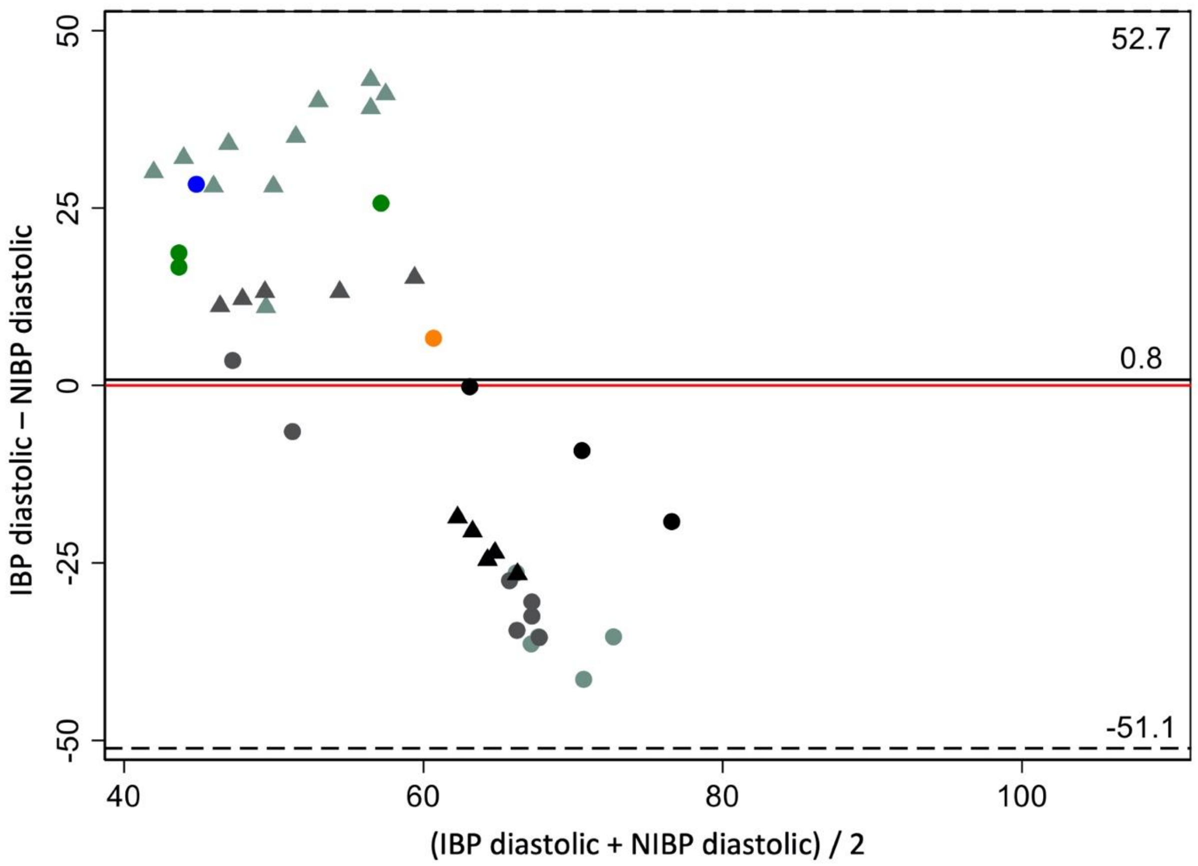

IBP invasive blood pressure, NIBP non-invasive blood pressure. *Bias* defines accuracy and *SD* defines precision.

Figure S3: Bland–Altman plots of agreement for 41 paired (a) systolic and (b) diastolic arterial blood pressure measurements (mm Hg) made non-invasively compared to those made invasively using the Bionet BM7Vet in 9 healthy, anaesthetised horses. The non-invasive arterial cuff was placed at the metacarpus. Repeated observations from the same horse are plotted using the same colour-shape combination. The solid black line indicates mean bias, and the dashed lines indicate lower and upper limits of agreement. The solid red line represents zero bias.

(a)

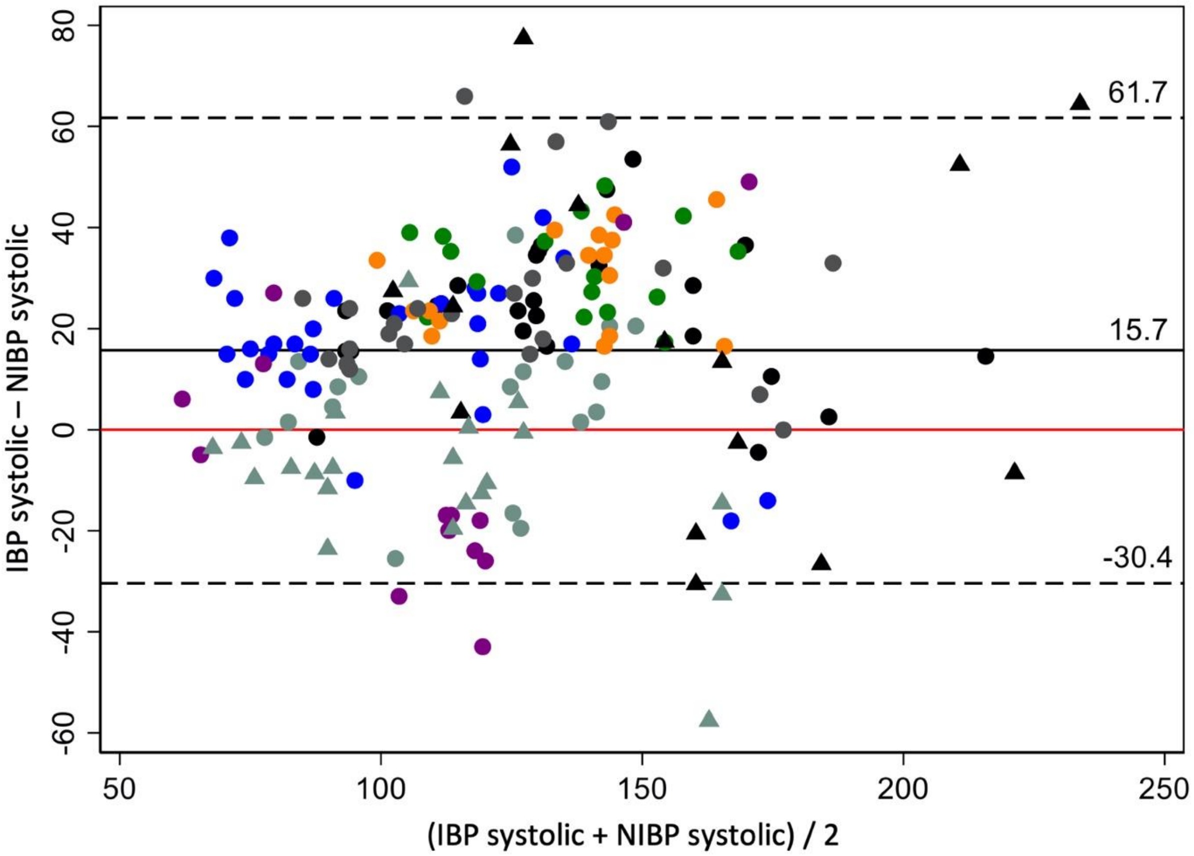

IBP invasive blood pressure, NIBP non-invasive blood pressure. *Bias* defines accuracy and *SD* defines precision.

(b)

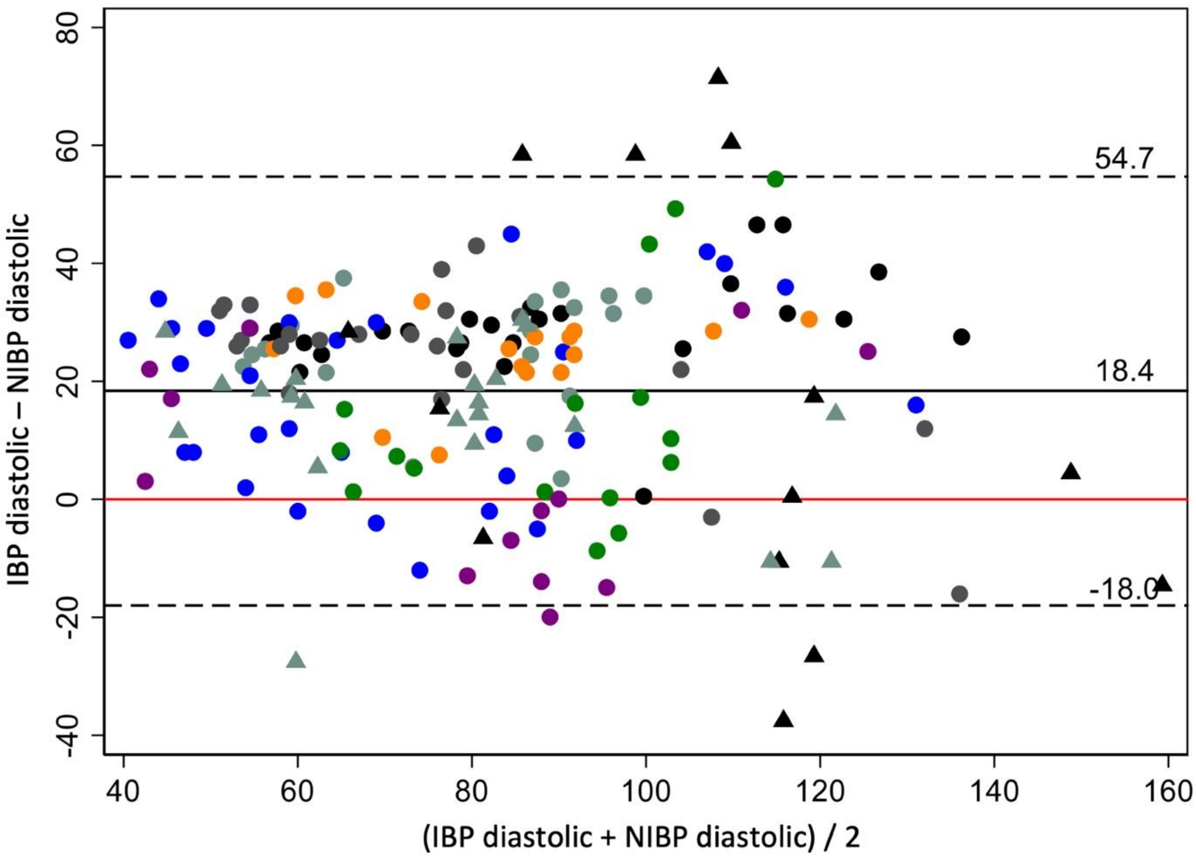

IBP invasive blood pressure, NIBP non-invasive blood pressure. *Bias* defines accuracy and *SD* defines precision.

Figure S4: Bland–Altman plots of agreement for 41 paired (a) systolic and (b) diastolic arterial blood pressure measurements (mm Hg) made non-invasively compared to those made invasively using the Bionet BM7Vet in 9 healthy, anaesthetised horses. The non-invasive arterial cuff was placed at the metacarpus. Repeated observations from the same horse are plotted using the same colour-shape combination. The solid black line indicates mean bias, and the dashed lines indicate lower and upper limits of agreement. The solid red line represents zero bias.
